# Physical activity is independently associated with reduced mortality: 15-years follow-up of the Hordaland Health Study (HUSK)

**DOI:** 10.1371/journal.pone.0172932

**Published:** 2017-03-22

**Authors:** Øyvind Kopperstad, Jens Christoffer Skogen, Børge Sivertsen, Grethe S. Tell, Solbjørg Makalani Myrtveit Sæther

**Affiliations:** 1 Department of Global Public Health and Primary Care, University of Bergen, Bergen, Norway; 2 Department of Health Promotion, Norwegian Institute of Public Health, Bergen, Norway; 3 Center for Alcohol & Drug Research, Stavanger University Hospital, Stavanger, Norway; 4 The Regional Centre for Child and Youth Mental Health and Child Welfare, Uni Research Health, Bergen, Norway; 5 Department of Research and Innovation, Helse Fonna HF, Haugesund, Norway; 6 Department of Clinical Science, University of Bergen, Bergen, Norway; Universiteit Hasselt, BELGIUM

## Abstract

**Background:**

Physical activity (PA) is associated with lower risk for non-communicable diseases and mortality. We aimed to investigate the prospective association between PA and all-cause and cause-specific mortality, and the impact of other potentially contributing factors.

**Method:**

Data from the community-based Hordaland Health Study (HUSK, 1997–99) were linked to the Norwegian Cause of Death Registry. The study included 20,506 individuals born 1950–1957 and 2,225 born in 1925–1927 (baseline age 40–49 and 70–74). Based on self-report, individuals were grouped as habitually performing low intensity, short duration, low intensity, longer duration or high intensity PA. The hazard ratios (HR) for all-cause and cause-specific mortality during follow-up were calculated. Measures of socioeconomic status, physical health, mental health, smoking and alcohol consumption were added separately and cumulatively to the model.

**Results:**

PA was associated with lower all-cause mortality in both older (HR 0.75 (95% CI 0.67–0.84)) and younger individuals (HR 0.82 (95% CI 0.72–0.92)) (crude models, HR: risk associated with moving from low intensity, short duration to low intensity, longer duration PA, and from low intensity, longer duration to high intensity). Smoking, education, somatic diagnoses and mental health accounted for some of the association between physical activity and mortality, but a separate protective effect of PA remained in fully adjusted models for cardiovascular (HR 0.78 (95% CI 0.66–0.92)) and respiratory (HR 0.45 (95% CI 0.32–0.63) mortality (both age-groups together), as well as all-cause mortality in the older age group (HR 0.74, 95%CI 0.66–0.83).

**Conclusion:**

Low intensity, longer duration and high intensity physical activity was associated with reduced all-cause, respiratory and cardiovascular mortality, indicating that physical activity is beneficial also among older individuals, and that a moderate increase in PA can be beneficial.

## Introduction

Physical activity is in principle both inexpensive and easily accessible, and may be a simple and cost-effective way to cut health care costs for society [1], improve quality of life [2], and prevent early death. Physical activity is frequently found to be associated with reduced all-cause mortality [3–6] and in particular mortality due to coronary heart disease [5, 7]. The inverse association between physical activity and mortality has been found in both men and women [3, 4, 8] and seems independent of a range of health-related factors including blood pressure, cholesterol levels, body mass index (BMI), diabetes, and smoking [4, 5]. It has also been suggested that physical activity reduces mortality risk independently of socioeconomic factors, such as education and income [9].

Both worldwide and in Norway [10], the major causes of death are non-communicable diseases, such as cardiovascular disease, cancer, diabetes and chronic respiratory disease [11]. The prevalence of non-communicable diseases is positively associated with health behaviours including physical inactivity, smoking, and harmful alcohol use. Factors such as high blood pressure, high blood sugar, high cholesterol and obesity, as well as mental distress and depression, are reported to be associated with increased risk of mortality [11]. Also low socioeconomic status is associated with increased all-cause and cause specific mortality, but to which extent this association can be explained by health behaviours remains unclear [12]. The separate risk factors could potentially have an impact on each other, and can, when combined, further increase the risk of disease and mortality. However, it is suggested that the total effect of having multiple risk factors are lower than the cumulative risk for the same individual factors [11].

Socioeconomic status and mental and physical health are also associated with health behaviours such as physical activity. There seems to be important differences in physical activity among population subgroups [13], and for instance, individuals living in areas of lower socioeconomic status are less likely to undertake vigorous activity [14]. Further, individuals with various mental (anxiety and depression [15], bipolar disorder [16] and physical (cardio-vascular disease [17], stroke [16, 18] and disabilities [19]) conditions, are less physically active than others. There is also evidence of clustering of risk factors for mortality and poor health, such as smoking, excess alcohol consumption and physical inactivity [20].

While we know that socioeconomic status, physical activity, other health behaviors and mental and somatic health are associated with mortality, less is known of the separete effect of physical activity after taking these other factors into account. The present study aimed to provide new insight into the independent protective effect of physical activity on mortality. Specifically, we aimed to investigate the associations between physical activity and all-cause and cause-specific mortality, and to investigate to what degree socioeconomic status, health-related behaviours (including smoking and alcohol consumption) and physical and mental health could explain potential associations between physical activity and mortality.

## Material and methods

### Population

The baseline data collection of the Hordaland Health Study (HUSK), Western Norway, was conducted during 1997–1999 as a collaboration between the Norwegian Health Screening Service, the University of Bergen and local health services [21]. Of the 38,587 invited, 25,532 (11,680 men and 13,852 women) accepted the invitation (66.2%), filled in questionnaires and attended clinical examinations including measures of blood pressure, height and weight. Among these, 18,481 were born in 1953–1957, 3,723 in 1950–51, and 3,328 in 1925–1927.

Individuals with missing information on physical activity (N = 490), education (N = 569), smoking (N = 1,531), alcohol consumption (N = 107), somatic diagnoses (N = 82), Body Mass Index (N = 37), blood pressure (n = 6), cholesterol (N = 33), and mental health (N = 650), in total (N = 2,801, 11.0%) were excluded, resulting in a study sample of 22,731 individuals.

### Variables

#### Outcome: Mortality

Data from HUSK were linked to the Norwegian Cause of Death Registry using the unique 11-digit identification number assigned to residents of Norway. The Cause of Death Registry contains information on cause of death for all deceased persons registered as residents in Norway at their time of death, whether death occurred in Norway or abroad [22, 23].

Participants were followed from the date they participated in HUSK (1997–1999) to death or end of follow-up (December 31, 2012), whichever occurred first. During the follow-up period from 1997 to 2012, in total 1,771 (6.9%) individuals died. In the 1925–27 group 36.5% of the excluded individuals and 36.8% of the included individuals died (p-value from chi^2^-test = 0.83). In the 1950–1957 group 3.2% of the excluded individuals and 2.4% of the included individuals died (p-value from chi^2^-test = 0.04).

To investigate cause-specific mortality, causes of death were grouped according to ICD-10 [24]. The largest groups, cardiovascular death (the I chapter), cancer deaths (the C chapter), respiratory deaths (the J chapter) and traumatic deaths (the V, W and X chapter) were analyzed.

#### Exposure: Physical activity

Participants reported how many hours of light physical activity (no sweating, not out of breath) and heavy physical activity (sweating, out of breath) they performed in an average week. The answer possibilities were 0, less than 1, 1–2, or 3 or more hours per week. This measure is used in CONOR (Cohort of NORway) [25], and has been shown to be correlated with relevant biological and anthropometric measurements [26]. As in a previous study [27] participants were divided into three groups based on total reported amount of physical activity. The groups are here named “*low intensity*, *short duration”*, *“low intensity*, *longer duration”* and *“high intensity “*. Low intensity, short duration applied to individuals who reported less than one hour light and less than one hour heavy training per week. Low intensity, longer duration applied to individuals who reported one or more hours light training and less than one hour heavy training per week. High intensity was defined as more than one hour heavy activity per week, regardless of the amount of light activity.

#### Sociodemographic variables

Marital status was grouped as *“Not married”*, *“Married/partner”*, *“Widow/widower”* or *“Divorced/separated”*. Participants reported their highest educational attainment in the following categories: *“Less than compulsory”*, *“Compulsory”*, *“High School (1–2 years)”*, *“High school”*, *“Less than 4 years in university”* and *“4 years or more in university”*. For baseline descriptive statistics education was presented in three groups *“Compulsory only”*, *“High school”* or *“University”*. For adjustment of the analyses investigating physical activity and mortality all categories were used.

#### Smoking and alcohol consumption

Participants were asked if they smoked pipe, cigars or cigarettes on a daily basis. Individuals answering yes to one or more of these were categorized as smokers (dichotomous variable).

Participants were asked; i) “Do you abstain from alcohol?” and ii) “How many glasses/drinks of alcohol do you normally consume in two weeks”. Based on this information, participants were divided into three groups. *“No consumption”* included those who reported to drink zero glasses/drinks in two weeks, as well as individuals reporting to abstain from alcohol and not providing information on number of drinks. As in previous publications *“moderate consumption”* included those having between one and 15 drinks per two weeks [27]. This group also included individuals reporting to not be abstainers, but who did not provide information on number of drinks. Individuals who reported a usual consumption of 16 or more drinks per two weeks were grouped in *“High alcohol consumption”*.

#### Physical health

Participants were asked whether they suffered from, or had previously suffered from myocardial infarction, angina pectoris, stroke, asthma, diabetes and/or multiple sclerosis. As in previous studies [27], the total number of diagnoses were captured in one variable with three categories; *“zero diagnoses”*, *“one diagnosis”*, and *“two or more diagnoses”*.

All participants attended a brief clinical examination. Weight and height were measured, and BMI was calculated. Blood pressure (diastolic and systolic) was measured three times and the mean values were used for analyses. Blood tests provided information on total cholesterol level measured in mmol/L.

#### Mental health

Participants’ mental health during the last two weeks was measured using the seven-item CONOR-Mental health index (CONOR-MHI) [28], which comprises 7 items assessing core symptoms of anxiety and depression. This CONOR-MHI is adapted from the Hospital Anxiety and Depression Scale (HADS) and the Hopkins Symptom Check List-10 (HSCL-10), and has previously shown acceptable psychometric properties [28]. Items included were: Have you, over the last two weeks, felt i) nervous and worried, ii) troubled by anxiety, iii) secure and calm, iv) irritable, v) happy and optimistic, vi) sad or depressed and vii) lonely. The answer possibilities for each item were one to four, with higher scores indicating poorer mental health. Individuals were included if answering at least one of the items. The mean score was used, and was attained by dividing the total sum score by seven, or the number of items answered.

### Statistical analyses

Baseline characteristics for both age groups were investigated according to level of physical activity using percentages for categorical variables and means with 95% confidence intervals (CI) for continuous variables. Differences between individuals performing different amounts of light and heavy physical activity were investigated using Pearson’s chi-squared tests and linear regression analyses.

Mortality risk was investigated using Cox proportional hazard models, stratified by age group and level of physical activity. Results are reported as hazard ratios (HR) with 95% CI. The physical activity variable had three levels, low intensity, short duration, low intensity, longer duration and high intensity, and could thus be used either continuously or categorically. In the initial analyses there were no statistical differences between low intensity, longer duration and high intensity physical activity in the risk of mortality when using low intensity, short duration activity as reference. The point estimates for high intensity compared to low intensity, longer duration physical activity were, however, consistently lower. In order to retain this information, we chose to present the results from the analysis were physical activity was entered as a three level continuous variable (assuming a linear trend). The HRs thus represents the risk associated with moving from low intensity, short duration to low intensity, longer duration PA, and from low intensity, longer duration to high intensity PA (the slope of the proportional hazard of mortality at any time point during follow-up).

The assumption of proportional hazards was investigated in all crude models. Cox regression models with Kaplan-Meyer estimates were also conducted using physical activity as an ordinal variable for old and young separately.

Cox proportional hazard models were also used to investigate the HR for cause specific mortality during follow-up. Due to the low numbers of deaths due to each cause in the younger group, these analyses were conducted on both groups combined.

Sensitivity analyses were performed, excluding individuals dying within the first year after participating in the HUSK baseline survey (N = 40, 15 among the younger and 25 among the older) from the analyses investigating both cause-specific and all-cause mortality.

In order to investigate the importance of socio-demographic variables, physical health (including cholesterol and blood pressure), mental health, as well as smoking and alcohol consumption, these variables were added to the Cox proportional hazards models, both separately and cumulatively. All multivariate analyses included adjustments for age and gender.

We also tested for an interaction effect between gender and physical activity on mortality by adding the interaction term to the models, but as no such interaction effects were observed (p-value form Likelihood ratio test = 0.74), all analyses were performed for both genders combined, adjusting for gender.

All analyses were conducted using STATA14 [29].

### Ethics

All participants gave their written consent at baseline. The current HUSK study was approved by the Regional Committee for Medical and Health Research Ethics of Western Norway (2015/2042/REK vest).

## Results

### Baseline characteristics

Among 20,506 individuals born during 1950–57 (53.3% women), 16.1% performed low intensity, short duration physical activity, 42.3% low intensity, longer duration physical activity, and 41.6% high intensity physical activity. As described in Table 1, 74.6% were married/had a registered partner, 64.0% reported high school or lower as their highest level of education and 36.8% smoked daily.

**Table 1:** Baseline variables among individuals aged 40–49 at baseline stratified by level of physical activity, the Hordaland Health Study.

*Born in 1950–1957*	Low intensity, short duration (16.1%)	Low intensity, longer duration (42.3%)	High intensity (41.6%)		All participants N = 20,506
	**Percentages**	**Percentages**	**Percentages**	p-value**	**Percentages**
*Deaths*	3.1% (n = 103)	2.5% (n = 214)	2.1% (n = 178)	0.004	2.4% (n = 495)
*Socio-Demographics*					
Female	46.4%	59.5%	49.7%	<0.001	53.3%
Marital Status				0.032	
*Not married*	12.4%	11.3%	12.2%		11.8%
*Married*	73.1%	75.3%	74.4%		74.6%
*Widow/widower*	0.6%	0.9%	0.6%		0.7%
*Divorced*	13.9%	12.5%	12.8%		12.9%
Education				<0.001	
*Compulsory only*	26.7%	18.9%	14.2%		18.2%
*High school*	46.9%	45.6%	45.6%		45.8%
*University*	26.4%	35.5%	40.1%		36.0%
*Health-Related Behaviour*					
Alcohol consumption				<0.001	
*No consumption*	34.6%	30.6%	23.3%		28.2%
*Normal consumption*	59.5%	64.3%	70.1%		65.9%
*Heavy consumption*	5.9%	5.1%	6.6%		5.9%
Daily smoking	47.4%	38.3%	31.1%	<0.001	36.8%
*Physical health*					
Somatic diagnosis				0.294	
*No diagnosis*	90.9%	91.8%	92.0%		91.7%
*One diagnosis*	8.5%	7.8%	7.6%		7.8%
*Two or more diagnosis*	0.6%	0.5%	0.4%		0.5%
	**Mean (SD)**	**Mean (SD)**	**Mean (SD)**	p-value***	**Mean (SD)**
Systolic blood pressure	128.7 (14.3)	127.7 (14.5)	128.0 (14.5)	0.129	128.0 (14.5)
Diastolic blood pressure	74.8 (10.5)	73.9 (10.5)	73.8 (10.4)	<0.001	74.0 (10.4)
Cholesterol (mmol/L)	5.70 (1.14)	5.60 (1.01)	5.54 (0.98)	<0.001	5.59 (1.02)
BMI	26.0 (4.2)	25.4 (3.9)	25.2 (3.5)	<0.001	25.4 (3.8)
*Mental health* *	1.62 (0.50)	1.54 (0.43)	1.49 (0.39)	<0.001	1.53 (0.43)

*7-item CONOR-MHI, mean score,

** p-values derived using chi^2^-tests,

*** p-values derived using linear regression

Among 2,225 individuals born 1925–1927 (49.2% women), 13.7% performed low intensity, short duration physical activity, 59.5% low intensity, longer duration physical activity, and 26.8% high intensity physical activity. Overall, 69.0% were married/had a registered partner, 81.4% reported their highest level of education to be high school or lower and 18.0% smoked daily. For more detailed baseline information within each level of physical activity, please refer to Table 2.

**Table 2:** Baseline variables among individuals aged 70–74 at baseline stratified by level of physical activity, the Hordaland Health Study.

Born in 1925–1927	Low intensity, short duration (13.7%)	Low intensity, longer duration (59.5%)	High intensity (26.8%)		All participants N = 2,225
	**Percentages**	**Percentages**	**Percentages**	p-value**	**Percentages**
*Deaths*	51.3% (n = 156)	35.2% (n = 466)	33.0% (n = 197)	<0.001	36.8% (n = 819)
*Socio-Demographics*					
Female	59.2%	53.2%	35.3%	<0.001	49.2%
Marital Status				0.059	
*Not married*	5.6%	5.7%	7.0%		6.1%
*Married*	65.8%	68.4%	72.0%		69.0%
*Widow/widower*	21.7%	20.6%	14.9%		19.2%
*Divorced*	6.9%	5.2%	6.0%		5.7%
Education				<0.001	
*Compulsory only*	60.2%	43.1%	27.1%		41.2%
*High school*	26.6%	40.6%	46.2%		40.2%
*University*	13.2%	16.2%	26.6%		18.6%
*Health-Related Behaviour*					
Alcohol consumption				<0.001	
*No consumption*	53.6%	47.3%	39.4%		46.0%
*Normal consumption*	43.8%	48.4%	53.8%		49.2%
*Heavy consumption*	2.6%	4.3%	6.9%		4.8%
Daily smoking	27.0%	17.5%	14.6%	<0.001	18.0%
*Physical health*					
Somatic diagnosis				0.001	
*No diagnosis*	58.2%%	70.4%	71.2%		68.9%
*One diagnosis*	29.0%	21.8%	20.6%		22.4%
*Two or more diagnosis*	12.8%	7.9%	8.2%		8.6%
	**Mean (SD)**	**Mean (SD)**	**Mean (SD)**	p-value***	**Mean (SD)**
Systolic blood pressure	148.3 (20.8)	147.6 (20.6)	146.9 (20.2)	0.330	147.5 (20.6)
Diastolic blood pressure	78.1 (11.9)	78.5 (12.4)	78.7 (11.6)	0.467	78.5 (12.1)
Cholesterol (mmol/L)	6.22 (1.19)	6.28 (1.14)	6.13 (1.06)	0.067	6.23 (1.13)
BMI	27.2 (4.8)	25.8 (3.8)	25.7 (3.3)	<0.001	26.0 (3.9)
*Mental health* *	1.67 (0.51)	1.54 (0.45)	1.47 (0.41)	<0.001	1.54 (0.45)

*7-item CONOR-MHI, mean score,

** p-values derived using chi^2^-tests,

*** p-values derived using linear regression

### Physical activity and all-cause mortality

During the 15-year follow-up period, a total of 1,314 (5.8%) individuals died; 2.4% in the 1950–57 group and 36.8% in the 1925–1927 group. Overall, a larger proportion of men than women died, 779 (7.3%) versus 535 (4.5%), respectively (p<0.001).

Physical activity was first investigated as a categorical variable, with mortality risk associated with low intensity, short duration physical activity as reference. In the younger age group, the crude HR of low intensity, longer duration physical activity compared to low intensity, short duration was 0.78 (95%CI: 0.62–0.99) and for hard compared to low intensity, short duration 0.66 (95%CI: 0.52–0.84). In the fully adjusted model the HR for low intensity, longer duration and high intensity physical activity were 0.96 (95%CI: 0.76–1.22) and 0.88 (95%CI: 0.69–1.13), respectively. In the older age group the crude HR was 0.60 (95%CI: 0.50–0.72) for low intensity, longer duration and 0.55 (95%CI: 0.45–0.68) for high intensity physical activity, and in the fully adjusted the HR was 0.64 (95%CI: 0.53–0.77) for low intensity, longer duration and 0.53 (0.42–0.66) for high intensity physical activity. As there were no significant differences between HR for low intensity, longer duration and high intensity physical activity, but consistently lower point estimates for high intensity physical activity, the rest of the results (apart from the Kaplan-Meier survival plot) show HR corresponding to the slope of mortality risk when physical activity was investigated as a three level continuous variable.

As described in Table 3, being physically active had a protective effect on total mortality for both the older and the younger age groups in crude models. In the younger age cohort, the HR for mortality was 0.82 (95% CI: 0.72–0.92; crude model). The HR did not materially change when adjusting for age and gender, but was no longer significant in the fully adjusted model (HR = 0.94, 95% CI: 0.83–1.06; adjusted for age, gender, marital status, alcohol consumption, smoking, BMI, cholesterol, systolic and diastolic blood pressure, somatic diagnoses and psychological distress). In the older cohort, the HR changed from 0.75 (95% CI: 0.67–0.84; crude model) to 0.74 (95% CI: 0.66–0.83) when fully adjusted.

**Table 3:** Hazard ratio for all-cause mortality associated with level of physical activity in two age groups, the Hordaland Health Study.

	*Participants born 1950–1957*, *baseline age 40–49*		*Participants born 1925–1927*, *baseline age 70–74*	
	*N = 20*,*506; 495 deaths*		*N = 2*,*225; 819 deaths*	
	HR for linear trend (95%CI)	P-value*	HR for linear trend (95%CI)	P-Value
Crude Model	0.82 (0.72–0.92)	**0.001**	0.75 (0.67–0.84)	**<0.001**
Adjusted for gender and age	0.82 (0.73–0.92)	**0.001**	0.68 (0.61–0.76)	**<0.001**
Adjusted for gender, age and marital status	0.82 (0.73–0.93)	**0.001**	0.68 (0.61–0.76)	**<0.001**
Adjusted for gender, age and education	0.86 (0.76–0.97)	**0.015**	0.69 (0.62–0.78)	**<0.001**
Adjusted for gender, age and alcohol	0.82 (0.73–0.92)	**0.001**	0.69 (0.61–0.77)	**<0.001**
Adjusted for gender, age and smoking	0.87 (0.77–0.98)	**0.021**	0.71 (0.64–0.80)	**<0.001**
Adjusted for gender, age and BMI	0.82 (0.73–0.93)	**0.002**	0.67 (0.60–0.75)	**<0.001**
Adjusted for gender, age and cholesterol	0.82 (0.73–0.93)	**0.001**	0.69 (0.61–0.77)	**<0.001**
Adjusted for gender, age and systolic BP	0.82 (0.73–0.92)	**0.001**	0.68 (0.61–0.76)	**<0.001**
Adjusted for gender, age and diastolic BP	0.82 (0.73–0.93)	**0.001**	0.68 (0.61–0.76)	**<0.001**
Adjusted for gender, age and somatic diagnoses	0.82 (0.73–0.93)	**0.001**	0.70 (0.63–0.79)	**<0.001**
Adjusted for gender, age and mental health**	0.86 (0.76–0.96)	**0.011**	0.70 (0.62–0.78)	**<0.001**
Fully adjusted model	0.94 (0.83–1.06)	0.290	0.74 (0.66–0.83)	**<0.001**

HR indicating the slope for risk associated with physical activity analyzed as a continuous variable with three levels; low intensity, short duration, low intensity, longer duration and high intensity. BMI: Body mass index, BP: Blood pressure

*p-values from cox-model

**7-item CONOR-MHI, continuous measure, mean score

**Bold:** statistically significant associations (p<0.05)

The test of proportional hazards gave no reason to reject the hypothesis that the hazards are proportional (crude model: chi^2^: 0.01, p = 0.92 for the younger group, chi^2^: 1.93, p = 0.16 for the older group). Sensitivity analyses excluding individuals who died during the first year of follow-up did not substantially change our results (all-cause mortality, fully adjusted; young: HR = 0.95, 95%CI: 0.84–1.07, old: HR = 0.74, 95%CI: 0.66–0.83).

### Physical activity and cause specific mortality

The most frequent cause of death was cancer (N = 520, 39.6% of 1,314), followed by cardiovascular and circulatory diseases (N = 344, 26.2%), trauma and accidents (N = 103, 7.8%), and respiratory disease (N = 89, 6.8%). When examining the effect of physical activity on the specific causes of death, a protective effect was observed for cardiovascular (HR = 0.78 (95% CI: 0.66–0.92)) and respiratory (HR = 0.45 (95% CI: 0.32–0.63)) mortality (fully adjusted). As detailed in Table 4, no protective effect was found for cancer or trauma and accidents.

**Table 4:** Hazard ratio for cause specific mortality associated with level of physical activity the Hordaland Health Study.

	*Cardiovascular diseases*		*Cancer*		*Respiratory diseases*		*Trauma /accidents*	
	HR for linear trend (95%CI)	P-value*	HR for linear trend (95%CI)	P-value*	HR for linear trend (95%CI)	P-value*	HR for linear trend (95%CI)	*P-Value
	*N = 344*		*N = 520*		*N = 89*		*N = 103*	
Crude Model	0.75 (0.63–0.88)	**<0.001**	0.90 (0.79–1.02)	0.096	0.45 (0.32–0.62)	**<0.001**	0.93 (0.70–1.22)	0.588
Adj. for gender and age	0.69 (0.59–0.81)	**<0.001**	0.89 (0.78–1.01)	0.068	0.42 (0.30–0.58)	**<0.001**	0.90 (0.69–1.18)	0.454
Marital status^x^	0.69 (0.59–0.81)	**<0.001**	0.89 (0.78–1.01)	0.072	0.41 (0.30–0.57)	**<0.001**	0.91 (0.69–1.19)	0.469
Education^x^	0.73 (0.62–0.86)	**<0.001**	0.90 (0.79–1.03)	0.128	0.42 (0.30–0.58)	**<0.001**	0.92 (0.70–1.21)	0.539
Alcohol^x^	0.69 (0.59–0.81)	**<0.001**	0.88 (0.77–1.00)	0.051	0.42 (0.30–0.58)	**<0.001**	0.91 (0.69–1.19)	0.486
Smoking^x^	0.72 (0.62–0.85)	**<0.001**	0.94 (0.83–1.07)	0.373	0.45 (0.33–0.63)	**<0.001**	0.95 (0.72–1.25)	0.706
BMI^x^	0.70 (0.59–0.82)	**<0.001**	0.88 (0.77–1.00)	**0.044**	0.39 (0.28–0.55)	**<0.001**	0.89 (0.68–1.17)	0.398
Cholesterol^x^	0.69 (0.59–0.81)	**<0.001**	0.88 (0.76–1.00)	0.054	0.42 (0.30–0.58)	**<0.001**	0.90 (0.69–1.18)	0.455
Systolic BP^x^	0.69 (0.59–0.82)	**<0.001**	0.88 (0.78–1.00)	0.058	0.41 (0.30–0.58)	**<0.001**	0.90 (0.69–1.18)	0.444
Diastolic BP^x^	0.69 (0.59–0.82)	**<0.001**	0.88 (0.78–1.01)	0.061	0.41 (0.30–0.58)	**<0.001**	0.90 (0.69–1.18)	0.461
Somatic diagnosis^x^	0.72 (0.61–0.84)	**<0.001**	0.89 (0.79–1.02)	0.084	0.45 (0.32–0.62)	**<0.001**	0.91 (0.70–1.19)	0.501
Mental health^x^**	0.71 (0.60–0.83)	**<0.001**	0.89 (0.79–1.02)	0.085	0.43 (0.31–0.60)	**<0.001**	1.00 (0.76–1.32)	0.988
Fully adjusted	0.78 (0.66–0.92)	**0.003**	0.94 (0.82–1.07)	0.359	0.45 (0.32–0.63)	**<0.001**	1.04 (0.79–1.38)	0.761

HR indicating the slope for risk associated with physical activity analysed as a continuous variable with three levels; low intensity, short duration, low intensity, longer duration and high intensity.

*From Cox model

^x^Also adjusted for age and gender.

BMI: Body mass index, BP: Blood pressure

**7-item CONOR-MHI, continuous measure

**Bold:** statistically significant associations (p<0.05)

Again, the test of proportional hazards gave no reason to reject the hypothesis that the hazards are proportional (crude models, cardiovascular, cancer and traumatic deaths respectively: chi^2^: 0.10, p-value: 0.754, chi^2^: 1.67, p-value: 0.196, chi^2^: <0.01, p-value: 0.974). For respiratory deaths, there were deviations from proportionality, chi^2^: 5.39, p-value: 0.020. These analyses were therefore run stratified by age group. In both age groups there was a protective effect of physical activity on respiratory death (young, fully adjusted model: HR = 0.24, 95%CI: 0.09–0.65, old, fully adjusted model: HR = 0.48, 95%CI: 0.33–0.70). In stratified analyses the assumption of proportional hazards was not violated (crude analyses: young: p-value = 0.348, old: p-value = 0.439).

Excluding individuals who died during the first year of follow-up did not substantially change our results (cause-specific mortality, both age groups together, fully adjusted: cardiovascular mortality: HR = 0.77, 95%CI: 0.65–0.91, cancer mortality: HR = 0.96, 95%CI: 0.84–1.09, respiratory mortality: HR = 0.45, 95%CI: 0.32–0.64, traumatic mortality: HR = 1.07, 95%CI: 0.81–1.42).

**Fig 1:**
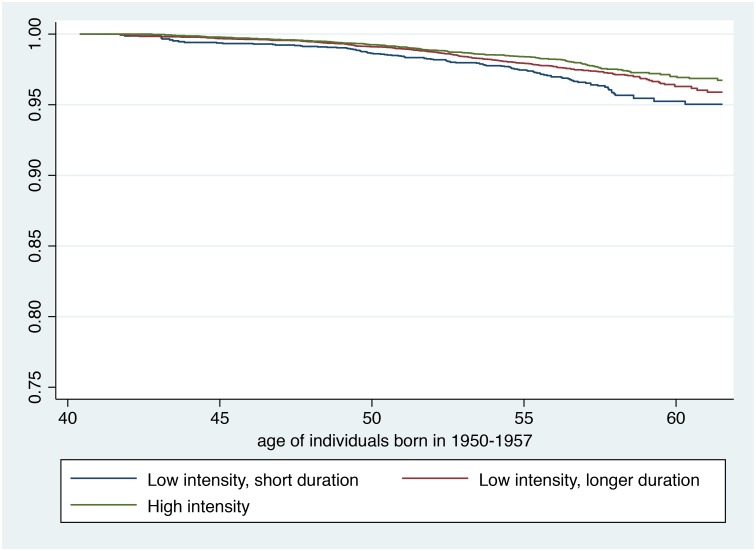
Kaplan-Meier survival estimates stratified by level of physical activity, individuals born in 1950–1957, the Hordaland Health Study.

**Fig 2:**
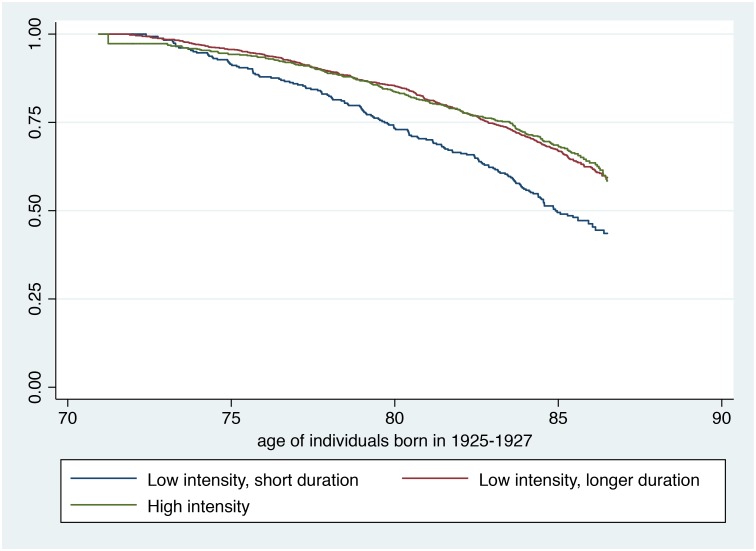
Kaplan-Meier survival estimates stratified by level of physical activity, individuals born in 1925–1927, the Hordaland Health Study.

Figs 1 and 2 display results from Kaplan-Meier survival model with physical activity analysed as a three-level ordinal variable. Fig 1 represents the younger age group, and Fig 2 the older age group. As the risk of dying was much lower in the younger age group, the y-axis on this fig was scaled to better fit the range. The figs show how physical activity is associated with increased survival, and that a protective effect of physical activity on mortality is not merely present among individuals performing a lot of high intensity physical activity, but also among those performing low intensity activity of longer duration.

### Other explanatory factors

As presented in Table 3, separate adjustment for potential confounding variables had little impact on the association between physical activity and mortality. In the younger age group, adjusting for smoking, education and mental health led to a 6%, 5% and 5% change in HR point estimate respectively (compared to the model adjusted for age and gender). In the older age group, adjusting for smoking, somatic diagnoses and mental health lead to a 4%, 3% and 3% change in HR point estimate respectively (compared to the model adjusted for age and gender). None of the adjustments led to significant changes–the 95%CI for HR point estimates after adjustment were overlapping with the crude 95%CI for all variables.

In the young age group, physical activity and mortality was no longer associated in the fully adjusted model. In the older age group, after adjustment for all included co-variables the HR point estimate was 9% higher than in the model adjusted for age and gender, and there was still a statistically significant protective effect of physical activity on mortality (HR: 0.74, 95%CI: 0.66–0.83).

Though attenuation achieved by controlling for separate confounding variables in this study in general were small, smoking, education, mental health and somatic diagnoses also explained some of the association between physical activity and cause-specific mortality (Table 4).

## Discussion

### Main findings

In this large, community-based study, physical activity was associated with reduced risk of all-cause mortality during a follow-up period of 15 years. There was a protective effect on mortality for both low intensity, longer duration and high intensity physical activity. Adjustment for separate potential confounding variables had little impact on the association between physical activity and mortality. The protective effect of physical activity on mortality was in the younger age group explained by other factors such as socioeconomic status, somatic and mental health, smoking and alcohol consumption. In the older age group, physical activity had an independent protective effect also in the fully adjusted model.

Physical activity was also associated with reduced risk of dying from cardiovascular and respiratory disease, but not from cancer or trauma/accidents. This effect on cardiovascular and respiratory mortality remained in the fully adjusted models

### Interpretations of findings

Our findings confirm previous research on a protective effect of physical activity on mortality [3–6]. The HR in the oldest group (after adjustments) is well in line with findings for individuals above 65 in a follow-up study based on different data material from the same Norwegian region [30]. A study of men from Oslo, Norway [31], and a study on physical activity and mortality among people with metabolic syndrome in Nord-Trøndelag, Norway, [32] suggest that the association may be even stronger.

In our study, physical activity was associated with reduced risk of dying from cardiovascular and respiratory disease, but not from cancer. Research on larger samples have found that higher pre-diagnosis leisure time physical activity is associated with lower risk of overall cancer mortality and mortality from multiple cancer sites [33]. Physical activity might also be associated with lower mortality risk among cancer survivors [34]. Our analyses included 520 cancer deaths. This might have provided too little power to detect a protective effect of physical activity on cancer mortality, in particular as etiology differs between specific cancer types.

The strong association between physical activity and mortality might be explained by several factors. It is known that physical activity has an impact on many health-related variables such as general fitness, hypertension, lung function, and blood sugar control [35]. Individuals who are physically active also seem to experience better mental health [36, 37], and our analyses show that some of the association between physical activity and mortality can be explained by mental health. One might suspect that high levels of physical activity could be a proxy measure for a healthy life-style in general. The development of many diseases, for instance cardiovascular disease, respiratory disease and some cancers is closely associated with lifestyle [11].

Our analyses show that for the older age group, somatic diagnoses explain part of the association between physical activity and mortality. It seems plausible that older individuals suffering from somatic diagnoses might not manage to be as physically active as others in their age group. At the same time, individuals in this age group who have been inactive for many years may have developed more somatic diagnoses due to inactivity. No matter the direction of association, both situations will contribute to an increased mortality risk. However, our study shows, in line with a previous study [38], that even when adjusting for physical health, a protective effect of physical activity on mortality remained. This indicates that physical activity can be beneficial also among less healthy elderly. This is further supported as physical activity seems to compensate for the increased mortality associated with low muscle strength [39]. Further, not just being active, but also taking up physical activity relatively late in life, can be associated with health benefits [40], further indicating that physical activity can be beneficial also among previously passive elderly.

Though most separate co-variables did not greatly affect the association between physical activity and mortality, education seemed to explain some of the association between physical activity and mortality in the younger age group, and in both groups, smoking seemed to be of importance. Educational attainment and mortality is closely linked worldwide [41, 42], and the association between mortality and smoking is strong and well-founded [43, 44]. Both smoking and low levels of education and physical activity might be indicators of an unhealthy lifestyle in general, and their separate effects are difficult to determine. However, our findings indicate that the positive effect of physical activity remains even after taking smoking into account. For the younger group the association of physical activity and mortality is not evident when adjusting for all available potential confounders, but in the older group, the effect remains in the fully adjusted model.

### Perspectives

The clear association between physical activity and reduced mortality found in our population-based study of two age groups imply that individuals aged 40 to 74 can all benefit from being physically active. Further, there seems to be a trend with more benefit accompanying more activity. However, the positive effect of physical activity was found not only among individuals performing high intensity physical activity but also among those performing low intensity, longer duration physical activity. Also previous research has indicated that there might be benefit also in moderate activity [45]. Knowing that increasing activity even just a bit could be beneficial might be motivating to individuals finding an extensive work-out program out of reach. Our observations further suggest that increasing the amount of physical activity performed in the population as a whole is likely to have a positive effect. Finding and reaching individuals who perform no or little physical activity should be a priority. Physical activity in some form is both cheap and simple, and it should be achievable for most people to reach the guidelines from the government, of 30 minutes of physical activity per day [46].

### Strength and limitations

The main strengths of this study lie in the large, population-based study design and the long follow-up time (15 years). We were able to examine risk of dying in relation to physical activity both in relatively young as well as older individuals. The questionnaire and the medical examination enable investigation of and adjustment for a broad range of potential explanatory variables.

The study has some notable limitations. The study design makes it impossible to determine causality. For instance, an individual suffering from a certain disease could be too sick to exercise and also at higher risk of dying. By adjusting our analyses for somatic diagnosis, as described in Tables 3 and 4, we aimed to at least in part take this into account. Sensitivity analyses were also conducted, and excluding individuals dying within the first year after participating in the HUSK baseline examination (N = 40, 15 in the younger age group and 25 in the older age group) did not substantially change our results.

Further, the number of cancer deaths might have been too low to find an effect of physical activity on cancer, and also cancer-type-specific analyses could be performed.

We aimed to investigate the separate effect of physical activity after taking physical health into account. This was done by adjusting analyses for systolic and diastolic blood pressure, BMI and cholesterol levels, as well as a count variable of number of somatic diagnoses reported. However, many other diagnoses than the included (myocardial infarction, angina pectoris, stroke, asthma, diabetes and/or multiple sclerosis) are of great importance for health and mortality risk. Not being able to include more diagnoses (an in particular cancer) is a limitation to the study. Also for other co-variables we expect the measures to not be ideal, and residual confounding cannot be ruled out.

Also sedentary behavior is associated with mortality [47, 48]. Not being able to include data on sedentary behavior in our analyses, or compare our level of physical activity with the ACSM guidelines, are limitations to the study.

We have previously shown that the individuals who were invited but declined to participate in HUSK, had poorer health than those who did participate [49]. We are thus investigating a sample somewhat healthier than if all individuals in the community had chosen to take part. Also, some individuals that participated did not provide all information of interest and were excluded from our analyses, and some differences were found between excluded and included. Among the older individuals, 36.5% of the excluded and 36.8% of the included died (p-value from chi^2^-test = 0.83), among the younger, 3.2% of the excluded and 2.4% of the included died (p-value from chi^2^-test = 0.04). Also with regards to physical activity there were some differences between those included and excluded (overall p<0.001, low intensity, short duration physical activity; 15.9% and 16.5% respectively, low intensity, longer duration; 44.0% and 49.9% respectively, and hard; 40.2% and 33.5% respectively). Such differences might lead to bias in our results. However, it has been argued that the risk of biased results is larger for prevalence estimates of exposures and outcomes than for exposure-outcome associations [50].

## Conclusion

In this population-based Norwegian study, both low intensity, longer duration and high intensity physical activity was associated with reduced all-cause mortality. In the older age group, an association remained in fully adjusted models. Physical activity was unassociated with cancer mortality, but was associated with reduced the risk of cardiovascular and respiratory mortality. Smoking, marital status, education, somatic diagnoses and mental health seemed to account for some of these associations, but PA retained a separate effect in fully adjusted models. Our results indicate that a moderate increase in physical activity can be beneficial, that the protective effect of physical activity is valid also after taking smoking into account, and that physical activity is beneficial also among older individuals.

## Abbreviations

ANOVA: Analysis of variance
BMI: Body Mass Index
BP: Blood Pressure
CI: Confidence Interval
CONOR-MHI: Cohort Norway-Mental health index
COPD: Chronic Obstructive Pulmonary Disease
HADS: Hospital Anxiety and Depression Scale
HUSK: Hordaland Health Study
HR: Hazard Ratio
HSCL-10: Hopkins Symptom Check List-10
PA: Physical activity
SES: Socioeconomic Status
